# National implementation of an optimal standardised technique for right-sided colon cancer: protocol of an interventional sequential cohort study (Right study)

**DOI:** 10.1007/s10151-023-02801-6

**Published:** 2023-04-25

**Authors:** Alexander A. J. Grüter, Usha K. Coblijn, Boudewijn R. Toorenvliet, Pieter J. Tanis, Jurriaan B. Tuynman, the Right Collaborators Group, Heiko Aselmann, Eric H.J. Belgers, Eric J.T. Belt, Stefan Benz, Roland S Croner, Peter van Duijvendijk, Jordan Fletcher, Christiaan Hoff, Roel Hompes, Danilo Miskovic, Anke B. Smits, Adam T. Stearns, Kristian E. Storli, Anthony W.H. van de Ven, Henderik L. van Westreenen

**Affiliations:** 1 Department of Surgery, Amsterdam UMC Location Vrije Universiteit Amsterdam, De Boelelaan 1117, Amsterdam, The Netherlands; 2 Cancer Center Amsterdam, Treatment and Quality of Life, Amsterdam, The Netherlands; 3 Department of Surgery, Antoni van Leeuwenhoek, Plesmanlaan 121, Amsterdam, The Netherlands; 4 Department of Surgery, Ikazia Hospital, Montessoriweg 1, Rotterdam, The Netherlands; 5 Department of Surgery, UMC Location University of Amsterdam, Meibergdreef 9, Amsterdam, The Netherlands; 6 Department of Surgery, Erasmus MC, Dr. Molewaterplein 40, Rotterdam, The Netherlands

**Keywords:** Right-sided colon cancer, Right hemicolectomy, Minimally invasive, Laparoscopic, Robot-assisted, National implementation

## Abstract

**Purpose:**

Minimally invasive right hemicolectomy (MIRH) is the cornerstone of treatment for patients with right-sided colon cancer. This operation has evolved during recent decades, with many innovations and improvements but this has also resulted in high variability of uptake with subsequent substantial variableness. The aim of this ongoing study is to identify current surgical variations, determine the most optimal and standardised MIRH and nationally train and implement that technique to improve short-term clinical and long-term oncological outcomes.

**Methods:**

The Right study is a national multicentre prospective interventional sequential cohort study. Firstly, current local practice was evaluated. Subsequently, a standardised surgical technique for right-sided colon cancer was determined using the Delphi consensus method, and this procedure was trained during hands-on courses. The standardised MIRH will be implemented with proctoring (implementation cohort), after which the performance will be monitored (consolidation cohort). Patients who will receive a minimally invasive (extended) right hemicolectomy for cT1-3N0-2M0 colon cancer will be included. The primary outcome is patient safety reflected in the 90-day overall complication rate according to the Clavien–Dindo classification. Secondary outcomes will include intraoperative complications, 90-day mortality rate, number of resected tumour-positive lymph nodes, completeness of mesocolic excision, surgical quality score, locoregional and distant recurrence and 5-year overall survival. A total number of 1095 patients (365 per cohort) will be included.

**Discussion:**

The Right study is designed to safely implement the best surgical practice concerning patients with right-sided colon cancer aiming to standardise and improve the surgical quality of MIRH at a national level.

**Trial registration:**

ClinicalTrials.gov: NCT04889456, May 2021.

**Supplementary Information:**

The online version contains supplementary material available at 10.1007/s10151-023-02801-6.

## Background

For colon cancer located in the caecum, ascending colon, hepatic flexure or proximal transverse colon, the cornerstone of treatment is minimally invasive right hemicolectomy (MIRH). This can either be performed by conventional laparoscopy or a robot-assisted technique. The aim of right hemicolectomy is to remove the right hemicolon with tumour-free margins and an intact mesentery containing all tumour-draining lymph nodes, to restore bowel continuity, and performing this procedure with the least possible surgical trauma. The disease-specific quality of MIRH is first of all determined by the extent and completeness of the mesocolic excision including the correct plane of dissection according to oncological principles. High-quality surgery aims to provide optimal locoregional control with minimal collateral damage, and will be reflected by the patient’s short- and long-term clinical outcomes [1–3]. As observed in clinical practice and noted in the literature, the surgical procedures are carried out with a high degree of variation between surgeons and institutions. In addition the quality of surgery is variable depending on learning curves, patient factors and availability of specialised teams. This variability is of relevance to clinical and oncological outcomes, thus illustrating the need for a standardised technique.

Substantial procedural variation in MIRH for right-sided colon cancer is present [4–6]. Minimal invasive surgery has been widely adapted, but the technique has been evolving during almost 30 years [7]. In the last decade, there has been specific focus on low-pressure pneumoperitoneum, intracorporeal anastomosis, extraction site and complete mesocolic excision (CME) technique. CME encompasses a dissection along the embryological planes to create an intact envelope of mesocolic fascia with a central vascular ligation to include all the draining lymph nodes along the supplying segmental vessels [8–12]. There is a disparity between current medical practice and the recommendations from best evidence and international guidelines regarding several steps of the MIRH, and there were also considerable regional differences [6]. These resemble the reality in surgical practice due to an absence of regular training programs, and a lack or a delay of implementation is present for the majority of innovations. Standardisation and subsequent training of surgical techniques and credentialing of surgeons have been found to reduce the adjusted in-hospital mortality [13, 14]. Recently, a large cohort study demonstrated that high surgical quality is associated with a better long-term survival of patients with colorectal cancer [15].

The current ongoing study aims to educate, train and implement a standardised minimally invasive surgical technique for right-sided colon cancer according to the latest and best evidence. The primary objective is to implement the new technique safely without an increase in the 90-day postoperative complication rate, while improving long-term oncological outcomes. The secondary objective is to validate a procedure-specific competency assessment tool (CAT) for this operation and use it to assess surgical quality to demonstrate the added value of training and proctoring and to correlate surgical quality with patient-related outcomes.

## Methods

This study protocol is written in accordance with the SPIRIT guidelines [16, 17]. The SPIRIT checklist is provided in the Appendix I in the supplementary material.

### Study design

The design of this trial is a national multicentre prospective interventional sequential cohort study including 43 participating centres (Appendix II in the supplementary material). Current local practice has been prospectively evaluated (and regarded/utilised as the first, control cohort) and subsequently compared to the intervention cohorts, after implementation of the optimal standardised surgical technique during a proctoring phase and then without intervening anymore in the consolidation phase (the second and third cohort respectively). A transition period is scheduled following the control cohort, in which consensus will be reached about the standardised MIRH using the Delphi method and education and hands-on training of the standardised technique is performed, before the technique will be implemented.

### Study objectives

The primary objective of this study is to safely implement a standardised technique of MIRH without increasing the 90-day postoperative complication rate, and to ultimately improve long-term outcomes for patients with right-sided colon cancer in the Netherlands. It is hypothesized that this will translate into a reduction of local and distant disease recurrence and thereby an improved 5-year overall survival (OS) and 3-year disease-free survival (DFS).

Secondary objectives are (1) to evaluate current national surgical variations of MIRH and their impact to clinical outcomes (already finished, namely phase 1); (2) to reach consensus concerning the standardised stepwise surgical technique for MIRH using the most recent insights from literature and guidelines and using the Delphi method (already finished) [18]; (3) to design and validate a CAT for the MIRH reflecting all the steps from the Delphi consensus; (4) to evaluate the effect of training and proctoring of the standardised MIRH (comparing control, implementation and consolidation cohorts) on surgical quality and (5) on short- and long-term clinical outcomes.

### Ethical consideration

The trial will be conducted according to Good Clinical Practice guidelines and the principles of the declaration of Helsinki (2013, [19]) and in accordance with the Medical Research Involving Human Subjects Act (WMO) and other guidelines, regulations and acts. This study is approved by the Medical Ethical Committee (METC) of the Amsterdam UMC, location VUmc (2021.0273). The protocol is registered by ClinicalTrials.gov (NCT04889456).

### Study population

Eligibility criteria for study participation include (1) patients with planned laparoscopic or robot-assisted (extended) right hemicolectomy for colon cancer of the caecum, ascending colon, hepatic flexure or proximal transverse colon, (2) age above 18 years and (3) signed informed consent.

A patient will not be eligible for inclusion in case of the presence of one or more of the following exclusion criteria: (1) cT4b/multivisceral resection, (2) cTNM stage 4 (M1), (3) ASA 4, (4) immune-modulating medication, (5) prior midline or transverse laparotomy larger than 10 cm (not including Pfannenstiel and McBurney’s incision), (6) perforated disease/peritumoral abscess/fistula, (7) acute obstruction, (8) emergency surgery, (9) neuroendocrine neoplasm (NEN), (10) other primary malignancy treated within 5 years from diagnosis of colon cancer, except for curatively treated prostate, breast, skin and cervical cancer.

### Informed consent procedure

Eligible patients will be approached for entry into the study at the first outpatient visit at the surgical department. The informed consent procedure includes study explanation, a written patient information sheet and adequate time for questions before signing the study consent form prior to the surgical procedure. Written informed consent is given by the patient to a participating surgeon, resident or research nurse at the outpatient clinic. Every included patient will be assigned a four-digit study number and only the local investigation sites have access to a decryption code.

### Study outline

**Fig. 1:**
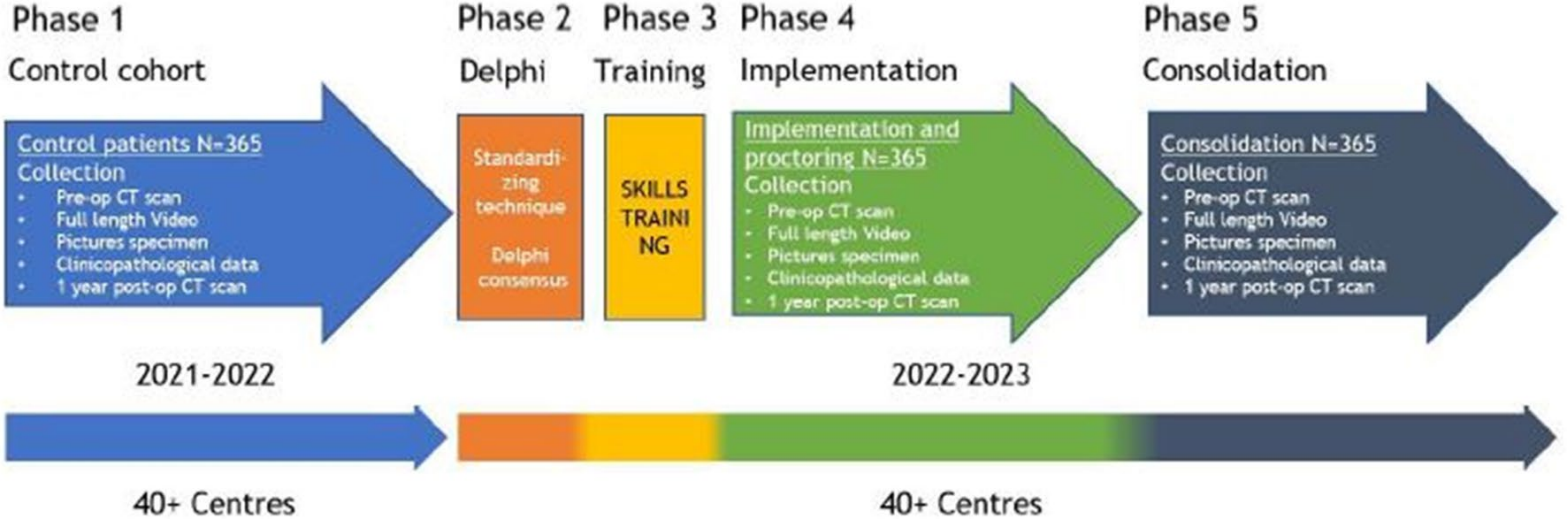
Schematic of the five-step approach of the Right study

This study consists of five phases as is schematically displayed in Fig. 1.

*Phase 1*: the variation of current surgical techniques amongst surgeons and centres for right-sided colon cancer was evaluated by prospective inclusion of consecutive patients into the *control cohort*. The first patient was included in October 2021, and currently all patients for this phase of the study have already been included. The preoperative CT imaging, full-length surgical video, pictures of the front and the back of the specimen and clinicopathological outcomes, as well as the CT imaging 1 year postoperatively (which is part of the routine oncological follow-up regimen in the Netherlands) were collected. The patients were treated according to the surgeon’s preference of surgical technique and local hospital perioperative care protocols. In this phase, anonymised video analysis of all the cases will be performed to evaluate variability and surgical quality.

*Phase 2*: a Delphi method was applied with two colorectal surgeons from each participating institution (*n* = 43) after the inclusion period of the control cohort was closed, to establish a detailed standardised technique of MIRH for colon cancer. The consented standardised technique includes essential elements that could potentially improve outcomes such as setup, surgical approach, mesocolic plane dissection, level of vascular ligation with the extent of vertical lymphadenectomy, the technique of the anastomosis and the extraction site.

*Phase 3*: the consented standardised technique was taught to all participating colorectal surgeons (*n* = 86) during a 1-day hands-on surgical *training* course. The standardised MIRH was trained in several skills centres in the Netherlands. The same two surgeons from each participating centre who contributed in the second phase participated in this phase. The courses consisted of a theory session with several presentations about the best evidence, anatomy and the optimal standardised surgical technique, followed by a hands-on training on human cadavers in the afternoon to apply and practise the technique. All proctors of the Right study first participated in a teach the teacher MIRH CME training in the presence of ESCP faculty before training the participating surgeons.

*Phase 4*: the new standardised MIRH technique will be implemented in a controlled fashion by proctoring the surgeons of the participating centres. The same two surgeons who were trained during phase 3 will consecutively include all eligible patients. This phase is referred to as the *implementation phase* and, similar to the control cohort, the preoperative CT imaging, full-length surgical videos, pictures of the front and the back of the specimen and clinicopathological outcomes as well as the CT imaging 1 year postoperatively will be collected. A total of 11 proctors throughout the Netherlands are selected on the basis of their experience in performing MIRH and in proctoring as well. They will be physically present within the operating room the first time the standardised technique is applied, after which teleproctoring will be used for the following sessions. Teleproctoring entails monitoring of the procedure by the proctor from a distance with the means of immediate communication between proctor and surgeon. After each proctor session, an evaluation (global assessment score, GAS) form will be completed in which the surgical procedure itself will be assessed on certain points and the specimen quality is rated. In addition, on that GAS form, both the proctor and the surgeon will each decide on the necessity to proctor/be proctored again. In phase 4, prospective anonymised video analysis of all the cases will be included with feedback to participating surgeons to stimulate the learning curve and to evaluate surgical quality.

*Phase 5*, the final phase: a third consecutive group of patients will be included to observe consolidation of the standardised technique after the learning curve without proctoring (*consolidation phase*). Again, the same preoperative CT imaging, full-length surgical videos, pictures of the front and the back of the specimen and clinicopathological outcomes as well as the CT imaging 1 year postoperatively will be collected and analysed, in order to be able to compare the performance with the control cohort and implementation period. This phase will involve a prospective and anonymous video analysis of every case to assess the surgical quality.

### Outcomes

The primary outcome of the current study is safety based on the overall 90-day postoperative morbidity rate, analysed and classified according to Clavien–Dindo [20]. Secondary outcomes include (1) intraoperative complications (i.e. vascular injury, injury to other organs), (2) 90-day mortality, (3) conversion rate, (4) operative time (minutes), (5) blood loss (millilitres), (6) validated assessment of the whole procedure using a CAT, (7) grading of the resection specimen according to Benz et al. [21], (8) total lymph node count in the specimen by the pathologist, (9) number of resected tumour-positive lymph nodes, (10) resection margins, (11) completeness of mesocolic excision based on the postoperative CT imaging (as performed 1 year postoperatively in regular oncological follow-up), (12) frequency of locoregional recurrence, (13) occurrence of distant metastasis, (14) 3-year DFS, (15) 5-year OS and (16) long-term surgical morbidity such as incisional hernia and adhesion-related small bowel obstruction.

### Sample size calculation

The sample size calculation is based on safety with non-inferiority for the primary endpoint, the overall 90-day morbidity. In the Dutch national colon cancer audit, the overall postoperative complication rate after MIRH was 26% [22]. As all contributing hospitals within this study are represented in the national database, it can be assumed that the control cohort of the Right study has the same percentage of postoperative complications, i.e. 26%. A non-inferiority margin of 7% was defined. Therefore, introducing the new standardised technique will still be considered safe if the overall postoperative complication rate does not exceed 33%. A total of 1095 patients (365 patient in each of the three prospective cohorts) will be needed to show that there is no difference between the control and experimental (i.e. standardised surgical technique) treatment. With this number of included patients, it can be demonstrated with 80% certainty that the lower limit of the one-sided 95% confidence interval (or equivalently a 90% two-sided confidence interval) will be above the non-inferiority limit of 7%.

### Statistical analysis

Baseline patient and procedure characteristics and perioperative outcome parameters are categorical, continuous and dichotomous variables, and will be presented accordingly. The chi-square test will be used to compare dichotomous and categorical data among patient groups. Descriptive outcomes will be reported as median with interquartile range (IQR) or mean with standard deviation (SD). According to their distribution, the Mann–Whitney *U* test will be used to evaluate intergroup variation.

Oncological outcomes will be determined using Kaplan–Meier analyses, with comparison of relevant patient groups using the log-rank test. Predictors of main outcome parameters will be determined by selecting relevant variables based on expected association with subsequent univariate analysis. Alongside the stratified comparisons as described above, multivariate analysis will be performed to determine the independent association of factors with a specific outcome parameter using logistic and Cox regression analyses. A *p* value of less than 0.05 will be considered to be statistically significant. All analyses will be performed with IBM SPSS statistics, version 23.00 (IBM Corp Amonk, NY, USA). Design of the CAT will be assessed to test reliability using generalizability theory. The outcome parameters of the CAT will be compared with clinical outcomes using univariate analysis and multivariate analysis to determine the independent association of factors with a specific outcome parameter using logistic and Cox regression analyses.

Before data is locked for analysis, the statistical analysis plan will be completed, and decisions will be made on stratification criteria, planned subgroup analysis, and how to handle baseline imbalance.

### Safety reporting

Owing to implementation of established surgical techniques within a controlled setting, the Right study is considered a low-risk study. As the patients in the control cohort will receive the standard of care, serious adverse events (SAEs) will not be reported. For the implementation and consolidation phases, SAEs will be documented up to 90 days following the operation.

The sponsor will immediately suspend the study if it is suspected, on the basis of sufficient signals, that continuation will jeopardise the health and/or safety of the subjects. The sponsor will notify the accredited METC without undue delay of a temporary halt including its reason. The study will be suspended pending a further positive decision by the accredited METC prior to continuation. The investigator will guarantee that all subjects are informed.

### Data handling and monitoring

Every included patient has been and will be assigned a four-digit study number. Communication occurs only with this number. The full name and date of birth of the patient have only been and will only be recorded on the informed consent form and these will be kept in a secure place in the participating hospitals, which is only accessible to anyone being officially involved in the Right study in that particular hospital.

In all participating hospitals, one of the surgeons acts as local principal investigator who is primarily responsible for execution of study interventions and for accuracy and completeness of the case report form (CRF). Data has been and will be digitally collected and stored using the electronic data management system Alea (https://www.aleaclinical.eu/). The digital platform has been specifically built for the purpose of the study by FormsVision, a privately owned business, which delivers clinical information technology solutions. This digital platform enables storage of full-length videos of surgical procedures, pictures of the specimen and CT imaging, and data entry for clinicopathological variables with all safety requirements concerning the privacy regulations. The design allows for secure worldwide online access to upload unlimited data 10 GB+ full-length videos and automatic consignments to reviewers by email with the ability to blindly assess the quality of surgery with a reviewer account.

### Public disclosure and publication policy

The Right study was registered at ClinicalTrials.gov (NCT04889456). Patients are entitled to public disclosure of the results of the study on the basis of their participation in it. The results of the study will be submitted for publication to peer-reviewed scientific journals regardless of the study outcomes, presented at international conferences and disseminated to relevant surgical and oncological associations. Participation agreements with respect to publication were completed prior to the start of the study. Authorship will be granted to all participants of the study group, and every other subject who made a substantial contribution to this study will be added to the collaborator list.

## Discussion

Ideally, the best surgical care for patients with right-sided colon cancer is available everywhere, independent of location, hospital or surgeon. Nevertheless, at present, many variations regarding surgical technique of MIRH are present, even in the Netherlands in which colorectal cancer care has been centralised during the last 20 years. This variation in technique is present between hospitals, but also between surgeons and is due to learning curves but mainly due to lack of implementation of recent innovations [23]. The aim of the present study is to identify the most optimal and standardised surgical technique for MIRH based on the best evidence in the literature and existing guidelines, to gain consensus amongst participating surgeons using a Delphi method, and thereafter to implement this standardised and consented procedure in a safe and controlled manner in the Netherlands. It is expected that this will result in the best, standardised procedure for patients with non-locally advanced right-sided colon cancer who are candidates for intentional curative segmental resection regardless of hospital or surgeon.

Multiple steps of MIRH and their variations can be identified, such as the extent and definition of lymph node dissection (D2 vs D3 lymphadenectomy), and the extent and definition of the CME concept, the type of anastomosis (e.g. intracorporeal or extracorporeal), the extraction site of the specimen (e.g. via a midline incision or via Pfannenstiel) and even smaller details such as use of oral antibiotics and the level of pneumoperitoneum. It is known from the literature that some variations have better clinical outcomes compared to others. The intra- and extracorporeal anastomoses have been extensively studied in MIRH for right-sided colon cancer and an intracorporeal anastomosis provides significantly better short-term outcomes regarding hospital stay, wound infection, time to first flatus and incision length [24–35]. Compared to alternative extraction sites, the Pfannenstiel incision is deemed superior owing to a much lower risk of incisional hernia [36–38]. The extent of the lymphadenectomy within the MIRH for colon cancer is currently a hot topic in the literature, however confusing. Many definitions of CME and D3 lymphadenectomy exist and both terms are incorrectly used as interchangeable concepts [39]. Within the Right study, both CME and D3 lymphadenectomy will be clearly defined within the process of achieving consensus on a standardised MIRH.

The primary outcome of the Right study, 90-day morbidity, was deliberately chosen in order to emphasize the need for a safe and controlled implementation of the optimal standardised technique. This is because any potential oncological benefit might be nullified in case of increased operative risks, which underlines the importance of safety first. If this goal has been achieved, we expect that the implementation and consolidation phases will demonstrate improved long-term oncological outcomes, with decreased recurrence rate and increased survival for patients with right-sided colon cancer in the Netherlands.

As a result of the risk of contamination in the control group when performing the Right study in a randomized controlled trial design, a prospective interventional sequential cohort study with a transition period was deemed the most appropriate design, in which routine practice will change in all centres at the same time after consensus has been reached and all surgeons have been trained. As mentioned in the “Methods” section, a transition period with training as well as a proctoring phase is included to increase safety and decrease the risk of any negative impact on clinical outcomes from the learning curve.

In many surgical trials where two procedures are compared, no surgical quality assessment (SQA) of the execution of the surgeries is performed. For example, one operation may seem to provide better clinical outcomes than another, but the quality of the surgical performance likely differs because of different levels of experience for the two procedures, which will inherently result in uncontrolled bias despite randomisation. To limit this type of bias, an operation-specific video-based CAT will be developed to evaluate the quality of each procedure [40]. The hypothesis is that a better score will also lead to better clinical outcomes for the patient. This has already been demonstrated in the literature [2, 41–43], and using an optimised CAT we will not only demonstrate the detailed quality with the different phases but also link the SQA scores to clinical outcomes which will deliver important insights which could be translated to many other surgical procedures.

If the results of the present study live up to expectations, this could influence future implementation of other procedures as well, because the same study setup can be applied for other disease entities. The five-phase approach, where firstly surgical variations are mapped, then the most optimal procedure is determined and consequently trained, then implemented safely with proctoring, and finally analysed on the basis of its performance after the learning curve, seems to be an ideal way to accomplish broad and safe implementation. The Delphi method in which all participating centres can take part will create a broad support base for the implementation of the new technique as every clinician is aware of its potential benefits in patient outcomes, and their knowledge and opinions are recognized in the creation of the ‘new’ standardised technique.

In conclusion, this study aims for safe implementation of an optimal standardised MIRH in all participating centres in the Netherlands, with the ultimate goal of improving long-term oncological outcomes. The design of the study deals with several important issues related to implementation of a new surgical procedure, and will provide valuable answers in how surgical quality can be improved on a large scale with reduction in unwanted practice variation, and how this will translate into better clinical outcomes for patients undergoing right-sided colon cancer surgery.

### Supplementary Information

Below is the link to the electronic supplementary material.

Supplementary file1 (DOCX 69 KB)

Supplementary file2 (DOCX 41 KB)

## Data Availability

My manuscript has no associated data. The gathering of data is ongoing. Data from the study will be published in separate scientific papers.
