# Supplementary material for: National implementation of an optimal standardised technique for right-sided colon cancer: protocol of an interventional sequential cohort study (Right study)
Source: Tech Coloproctol. 2023 Apr 25;27(11):1083–90. doi: 10.1007/s10151-023-02801-6 (PMC10562307; doi:10.1007/s10151-023-02801-6)
Supplement: Supplementary file 2 — Supplementary file2 (DOCX 41 KB) [file 10151_2023_2801_MOESM2_ESM.docx]

**Supplementary material file II: Participating centres Right study**

1. Admiraal de Ruyter Hospital, Vlissingen
2. Albert Schweitzer Hospital, Dordrecht
3. Alrijne Hospital, Leiderdorp, Leiden, Alphen aan den Rijn
4. Amstelland Hospital, Amstelveen
5. Amsterdam UMC, location AMC, Amsterdam
6. Amsterdam UMC, location VUmc, Amsterdam
7. Antoni van Leeuwenhoek, Amsterdam
8. Beatrixhospital Rivas Zorggroep, Gorinchem
9. Bravis Hospital, Bergen op Zoom/Roosendaal
10. Canisius Wilhelmina Hospital, Nijmegen
11. Deventer Hospital, Deventer
12. Diakonessenhuis Utrecht, Utrecht
13. Dijklander Hospital, Hoorn/Purmerend
14. Elisabeth-TweeSteden Hospital, Tilburg
15. Flevoziekenhuis, Almere
16. Franciscus Gasthuis Vlietland, Rotterdam
17. Gelre Hospitals, Apeldoorn, Zutphen
18. Groene Hart Hospital, Gouda
19. Haaglanden Medical Center, Den Haag
20. Hospital Gelderse Vallei, Ede
21. Hospital Groep Twente, Hengelo, Almelo
22. Hospital St Jansdal, Harderwijk
23. IJsselland Hospital, Capelle aan den IJssel
24. Ikazia Hospital, Rotterdam
25. Isala Hospitals, Zwolle, Meppel, Kampen, Steenwijk, Heerde
26. LangeLand Hospital, Zoetermeer
27. Laurentius Hospital, Roermond
28. Maastricht University Medical Center, Maastricht
29. Maasziekenhuis Pantein, Beugen
30. Meander Medical Center, Amersfoort
31. Medical Center Leeuwarden, Leeuwarden
32. Nij Smellinghe, Drachten
33. Onze Lieve Vrouwe Gasthuis, Amsterdam
34. Rijnstate Hospital, Arnhem
35. Rode Kruis Hospital, Beverwijk
36. Sint Jansgasthuis, Weert
37. Spaarne Gasthuis, Haarlem, Hoofddorp
38. Stichting ZorgSaam Zeeuws Vlaanderen, Terneuzen
39. St Antonius, Niewegein
40. Van Weel-Bethesda Hospital, Dirksland
41. VieCuri Medical Center, Venray
42. Zaans Medical Center, Zaandam
43. Zuyderland Medical Center, Heerlen, Sittard-Geleen
